# Self-Acne Treatment Among Medical Field Students at Qassim University

**DOI:** 10.7759/cureus.91820

**Published:** 2025-09-08

**Authors:** Mohammed Albeshri

**Affiliations:** 1 Department of Dermatology, College of Medicine, Qassim University, Buraydah, SAU

**Keywords:** acne, acne vulgaris, dentists, pharmacology, pimples, self-care, self-medication, stress, topical treatment

## Abstract

Background

Acne vulgaris is a common inflammatory condition affecting the pilosebaceous units of the skin, with a significant impact on adolescents and young adults. Despite the availability of professional treatments, self-medication practices are prevalent, especially among medical students with easy access to medications and pharmacological knowledge. This study aims to assess the prevalence of self-treatment for acne among medical field students at Qassim University, Saudi Arabia, and to explore their attitudes, practices, and knowledge regarding self-medication.

Materials and methods

A cross-sectional study was conducted at Qassim University between July and August. An anonymous web-based questionnaire comprising 27 items was distributed to students aged 18 years or older. The survey collected data on demographics, acne presence, self-treatment practices, medication types, and attitudes towards self-medication.

Results

Of the 180 participants, 63.3% reported self-treating their acne, with females (37.8%) engaging in self-medication more than males (25.6%) (p = 0.003). The face was the most commonly affected area (38.9%). Topical treatments such as tretinoin and benzoyl peroxide were the most frequently used medications, while cleansers were the most common over-the-counter products. Self-medication was driven by perceptions of mild disease severity (23.3%) and easy accessibility to treatments (20%). Notably, 52.2% engaged in self-picking behavior, and while 80.6% believed acne warranted a dermatologist’s consultation, many still relied on personal judgment or social media for treatment decisions.

Conclusion

Self-treatment of acne is highly prevalent among medical students, influenced by perceived disease mildness, accessibility of medications, and pharmacological knowledge. While many students demonstrated awareness of drug dosages and adverse effects, the lack of professional oversight poses risks of treatment failure, skin complications, and psychological distress. These findings highlight the need for educational interventions to promote evidence-based acne management and discourage risky practices such as self-picking. Encouraging students to seek dermatological care and raising awareness of the potential consequences of unsupervised self-treatment are essential for optimizing skin health and overall well-being.

## Introduction

Acne vulgaris is a condition characterized by inflammation of the pilosebaceous units in the skin, predominantly affecting the face and trunk. Acne vulgaris impacts around 9% of the global population, with a particularly high prevalence among adolescents and young adults, affecting approximately 85% of individuals aged 12 to 24 years [1].

The prevalence of acne increased significantly with age, peaking in late adolescence, then gradually decreasing as people got older. Acne is uncommon in individuals over 50. In teenagers and young adults, males are more likely to have acne, whereas in those over 30, females are more likely to have acne. Among individuals with acne, the majority had mild cases, with fewer having moderate to severe forms. Persistent acne is much more common in adults than acne that starts later in life. Additionally, smoking and alcohol consumption are linked to acne in teenagers [2].

Acne lesions typically affect the pilosebaceous follicles and are influenced by interconnected processes such as sebum production, colonization by *Cutibacterium acnes* (which was known as *Propionibacterium acnes*), and inflammation [3]. One widely used scale for assessing acne severity is the Global Acne Severity Scale, which was developed by Dréno et al. [4]. This scale was validated through photographic analysis. Also, this scale focuses on facial acne, rating severity from 0 to 5 based on the number and type of lesions present. Other frequently used classification systems include the Global Alliance classification, which categorizes acne into five types based on severity: mild comedonal, mild papulopustular, moderate papulopustular, moderate nodular, and severe nodular [5]. Additionally, the European guidelines propose a four-level scale: comedonal, mild-to-moderate papulopustular, severe papulopustular and moderate nodular, and severe nodular and conglobate [6].

Self-medication involves using drugs to address disorders or symptoms that one has diagnosed themselves. One significant drawback of self-medication is the absence of a clinical assessment by a qualified healthcare professional, which can lead to misdiagnosis and delays in receiving appropriate treatment [7]. Self-medication can manifest in various ways, including acquiring medication without a prescription, sharing medications with others, or using drugs that are already available at home [8]. Medical students often engage in self-medication, reaching a prevalence of 52.2%, influenced by factors such as easy access to medications, their exposure to medical environments, and their understanding of pharmacology. While the World Health Organization (WHO) supports self-medication for minor health issues, it also warns about potential risks, including adverse side effects [9].

This study aims to determine the prevalence of self-acne treatment among medical field students at Qassim University, Saudi Arabia, and assess the self-medication of acne cases among this population group.

## Materials and methods

Inclusion and exclusion criteria

Participants eligible for inclusion in this study were male or female university students aged 18 years or older, who were able to read and understand Arabic and who provided informed consent before participation. Students who did not meet these criteria - either due to age below 18, lack of Arabic language comprehension, or failure to provide informed consent - were excluded from the study. No additional exclusion criteria were applied.

Sampling techniques and study design

This study employed a cross-sectional design to assess the prevalence of acne and self-medication practices among university students. Data collection was performed using a self-administered, anonymous, web-based questionnaire, which included 27 items grouped into several domains: demographic characteristics (e.g., age, gender, academic level), presence and site of acne, experience with self-treatment, and attitudes and practices related to self-medication.

The questionnaire was disseminated electronically via social media platforms (e.g., WhatsApp, Twitter, and Telegram) commonly used by university students, allowing for broad and voluntary participation. Participation was entirely voluntary, and informed consent was explicitly obtained at the beginning of the survey form. The online format ensured anonymity and facilitated data collection across various departments and colleges within the university.

Study location and duration

The research was conducted at Qassim University, located in Qassim State, Saudi Arabia. The data collection period spanned two months (from July 1 to August 31, 2024).

Ethical approval

The study protocol was reviewed and approved by the Research Ethics Committee of the Qassim Health Cluster. Ethical approval was granted under IRB approval number: QHC-IRB-2024-038, dated June 24, 2024. All participants were informed about the study objectives, confidentiality of responses, and their right to withdraw at any time without any consequences.

Statistical analysis

As the study was primarily descriptive, no formal sample size calculation was performed. However, data from all eligible and consenting participants were included in the analysis to maximize statistical power and generalizability.

Descriptive statistics were used to summarize the demographic and clinical characteristics of the participants. The chi-square test (χ²) was applied to examine the association between categorical variables, such as gender or academic year, and the presence of acne or self-medication behaviors. A p-value of <0.05 was considered statistically significant. All analyses were conducted using IBM SPSS Statistics software, Version 25.0 (IBM Corp., Armonk, NY, USA).

## Results

Characteristics of study subjects

The study included a total of 180 medical field students, evenly split by gender, with 90 females (50.0%) and 90 males (50.0%). The majority of participants were aged 19-23 years, comprising 135 (75.0%) students, while the remaining 44 (24.4%) students were aged 24-27 years. In terms of educational phases, 46 (25.6%) students were in the pre-clinical phase, 86 (47.8%) students were in the clinical phase, and 23 (12.8%) students were in their internship year. Additionally, there were 16 pharmacy students or interns (8.9%) and eight dental students or interns (4.4%). Regarding personal experience with self-treatment of acne, 114 (63.3%) students reported having self-treated their acne. Detailed characteristics of study subjects are shown in Table 1.

**Table 1:** Characteristics of study subjects

Total = 180		Frequency (n)	Percentage (%)
Gender	Female	90	50.0
	Male	90	50.0
Age group	19–23	135	75.0
	24–27	44	24.4
Education	Pre-clinical phase	46	25.6
	Clinical phase	86	47.8
	Internship year	23	12.8
	Pharmacy (student/intern)	16	8.9
	Dental (student/intern)	8	4.4
Personal experience with self-treatment of acne	Yes	114	63.3
	No	64	35.6

Characteristics of acne patients with self-treatment experience

Among the participants, 68 (37.8%) females reported having self-treated their acne, compared to 21 (11.7%) females who did not. In contrast, 46 (25.6%) males engaged in self-treatment, while 43 (23.9%) males did not. Overall, a majority of the students, 114 (63.4%), had experience with self-acne treatment, whereas 64 (35.6%) had no experience (p = 0.003).

Among students aged 19-23 years, 86 (47.8%) reported engaging in self-treatment for acne, while 47 (26.1%) did not; in the age group of 24-27 years, 27 (15.0%) reported self-treatment (p = 0.884).

In the pre-clinical phase, 33 (18.4%) students reported self-treating their acne. During the clinical phase, 49 (27.2%) students engaged in self-treatment, while 36 (20.0%) students did not. Among those in their internship year, 18 (10.0%) students practiced self-treatment. For pharmacy students and interns, the numbers were equal, with eight (4.4%) in both the self-treatment and non-self-treatment groups. Similarly, in the dental field, six students or interns (3.3%) reported self-treatment, compared to 2 (1.1%) who did not (p = 0.049) (Table 2).

**Table 2:** Characteristics of acne patients with self-treatment experience

		Yes (self-treatment of acne)	No (self-treatment of acne)	χ² test	P-value
Gender	Female	68 (37.8%)	21 (11.7%)	11.808	0.003
	Male	46 (25.6%)	43 (23.9%)		
Age (years)	19-23	86 (47.8%)	47 (26.1%)	1.403	0.884
	24-27	27 (15.0%)	17 (9.4%)		
Educational phase	Pre-clinical phase	33 (18.4%)	12 (6.7%)	26.376	0.049
	Clinical phase	49 (27.2%)	36 (20.0)		
	Internship year	18 (10.0)	5(2.9)		
	Pharmacy (student/intern)	8 (4.4)	8 (4.4)		
	Dental (student/intern)	6 (3.3%)	2 (1.1%)		
Total		114 (63.4%)	64 (35.6%)	-	-

Site of acne

The majority of students reported experiencing acne on the face, with 70 (38.9%) individuals affected. A smaller proportion had acne on both the face and back (14 students, 7.8%), and a notable number had acne on the face, back, and chest simultaneously (24 students, 13.3%. Table 3 shows the prevalence of acne according to the body site.

**Table 3:** Site of acne

		Frequency	Percentage
Site of acne	Face	70	38.9%
	Chest	0	0.0%
	Face and chest	2	1.1%
	Back	3	1.66%
	Face and back	14	7.8%
	Chest and back	1	0.55%
	Face, and back and chest	24	13.3%

Self-medication for acne

The most commonly used medications were topical tretinoin and benzyl peroxide, with 49 and 47 students using them, respectively. While doxycycline, topical azelaic acid, and isotretinoin were each used by 24 students, topical adapalene was chosen by 22 students. Topical clindamycin was used by 19 students, and anti-androgens (spironolactone) were the least used, with only one student reporting its use.

Among the respondents, 78 participants reported using only a single medication, while 55 participants used two or more medications. Additionally, 47 participants did not select any medication.

Most commonly used over-the-counter treatments

Regarding the common types of over-the-counter (OTC) medications used at home, the results indicated that cleansers were the most frequently used, with 108 students reporting their use. Leave-on products were the next most common, used by 42 students. Other treatments included vitamins (26 students), home remedies and herbal (24 students), essential oils (18 students), mechanical treatments (16 students), and peels (15 students).

Among the participants, 65 students reported using only a single OTC medication, while 75 students used two or more medications. Additionally, 40 students did not select any OTC medication.

Reasons for self-treatment of acne

The most common reason was the perception that the disease severity was mild, reported by 42 (23.3%) students. Easy accessibility to treatments was the second most cited reason, with 36 (20%) students indicating this factor. Pharmacology knowledge also played a significant role, influencing 27 (15.0%) students to self-medicate. Additionally, 42 (23.3%) students did not specify their reasons. The reported reasons for self-treatment are shown in Table 4.

**Table 4:** Reasons for self-treatment of acne

Reasons for self-treatment of acne	Frequency	Percentage
Disease severity is mild	42	23.3
Easy accessibility	36	20
Know the treatment prescribed previously	15	8.3
Feeling embarrassment when discussing symptoms	3	1.7
Insufficient time	10	5.6
Pharmacology knowledge	27	15.0
Missing	42	23.3
total	180	100.0

The most frequently cited information sources were drug advertisements and social media, reported by 42 (23.3%) students. Decision-making based on personal judgment was also significant, with 30 (16.7%) students relying on this method. Family and friends, as well as pharmacists, were each mentioned by 22 (12.2%) students as sources of information. Books were a source for 17 (9.4%) students. Table 5 describes the frequency of reported sources of information for self-treatment behavior.

**Table 5:** Sources of information

Sources of information	Frequency	Percentage
Family and friends	22	12.2
Prescriptions written for others	4	2.2
Decision-making	30	16.7
Drug advertisements and social medial	42	23.3
Books	17	9.4
Pharmacist	22	12.2
Leftover drug	3	1.7
Missing	40	22.2

Self-picking of acne pimples

A significant portion of participants, 94 (52.2%), engaged in self-picking (squeezing) of acne. In comparison, 72 (40.0%) reported not engaging in this behavior. According to the necessity of squeezing acne, the majority of participants, 144 (80.0%), believed it was not necessary, while only 22 (12.2%) considered it necessary. Table 6 describes the prevalence of self-picking behaviors.

**Table 6:** Self-picking of acne pimples

		Frequency	Percentage
Self-picking of acne (squeezing)	Yes	94	52.2
	No	72	40.0
	Missing	14	7.8
Squeezing is necessary	Yes	22	12.2
	No	144	80.0
	Missing	14	7.8

Students’ knowledge of self-medication

A significant portion of students demonstrated awareness of the dosage of the drug, with 91 (50.6%) students answering affirmatively. Knowledge about the mode of action was reported by 87 (48.3%) students, while 94 (52.2%) students were aware of potential adverse drug reactions. Precautions for use were known by the majority, with 110 (61.1%) students indicating awareness. Additionally, 96 (53.3%) students understood the potential complications associated with self-medication. The student's knowledge regarding self-medication is described in Table 7.

**Table 7:** Students’ knowledge towards self-medication

Students’ knowledge towards self-medication	Yes (%)	No (%)
Dosage of the drug	91 (50.6)	53 (29.4)
Mode of action	87 (48.3)	62 (34.4)
Adverse drug reaction	94 (52.2)	55 (30.6)
Precautions for use	110 (61.1)	38 (21.1)
Complication	96 (53.3)	53 (29.4)

Regarding the statement of self-picking behavior, 37 (20.6%) of the participants reported that it is difficult to stop once the picking or squeezing is started, while 26 (14.4%) attributed their actions to self-consciousness about appearance. Table 8 gives a description of self-picking behavior of acne pimples.

**Table 8:** Reasons for self-picking of acne pimples

Statement of participating in self-picking of acne (squeezing)	Frequency	Percentage
I often pick or squeeze my acne pimples until they start to bleed	11	6.1
I tend to squeeze or pick my acne pimples whin I am under stress	19	10.6
I tend to squeeze or pick my acne pimples whin I feel self-conscious about my appearance	26	14.4
Once I start picking or squeezing my acne pimples. It is difficult for me to stop.	37	20.6
Missing	30	16.7

Isotretinoin use

Among the participants, 32 (17.8%) students reported using isotretinoin for their acne treatment, while a significant majority, 122 (67.8%) students, did not use isotretinoin. Additionally, 26 (14.4%) students did not respond. When asked about conducting initial laboratory testing before starting isotretinoin, the same distribution was observed: 32 (17.8%) students confirmed they had done the testing, 122 (67.8%) students had not, and 26 (14.4%) students did not respond. Table 9 describes the prevalence of isotretinoin use among the participants.

**Table 9:** Isotretinoin use

Is isotretinoin among the option you used to treat your acne by your own	Frequency	%
Yes	32	17.8
No	122	67.8
Missing	26	14.4

Surprisingly, isotretinoin use in 122 (67%) of the participants was not preceded by laboratory tests, in comparison to only 32 (17.8%) who started using the drug after necessary laboratory tests, as shown in Table 10.

**Table 10:** Laboratory testing before starting isotretinoin

Did you do the initial laboratory testing before starting isotretinoin?	Frequency	%
Yes	32	17.8
No	122	67.8
Missing	26	14.4

Students’ attitude and practice towards self-medication

Among medical field students’ attitudes and practices towards self-medication, a significant portion of students (145, 80.6%) believe that acne requires a dermatologist’s consultation, and 143 (79.4%) students consider follow-up for acne important. However, only 60 (33.3%) students encourage friends and family to self-medicate, while a majority (103, 57.2%) do not. When it comes to practical habits, 109 (60.6%) students frequently read the directions on a drug label, and 91 (50.6%) students check the expiration date when purchasing a drug. Additionally, 66 (36.7%) students reported that medications are available at home or hostel at all times. Notably, 123 (68.3%) students view self-medication as a component of self-care. Table 11 shows the students' attitude and practice towards self-medication.

**Table 11:** Students’ Attitude and Practice towards Self-Medication Students’ attitude and practice towards self-medication

Students’ attitude and practice towards self-medication	Yes (%)	No (%)
Encourage friends and family to self-medicate	60 (33.3)	103 (57.2)
Does acne require a dermatologist’s consultation?	145 (80.6)	23 (12.8)
Is follow-up for acne important?	143 (79.4)	25 (13.9)
How often do you read the directions on a drug label?	109 (60.6)	42 (23.3)
When purchasing a drug, do you check its expiration date?	91 (50.6)	59 (32.8)
Are the medications available at home/hostel at all times?	66 (36.7)	87 (48.3)
Self-medication is a component of self-care	123 (68.3)	38 (21.1)

## Discussion

Literature review

A cross-sectional study was conducted by Al Robaee to investigate the prevalence of acne among students at Qassim University, as well as to assess their knowledge, beliefs, and the psychological impact of acne on them. A total of 717 students (381 males and 336 females) were observed at the university’s medical clinics. The study found that 56.2% of the students had acne, with no significant difference between genders. Nearly half of the participants had been dealing with acne for over a year. Among those who sought medical advice, 40.3% did so within three months of onset, and 58.9% sought help on their own initiative. It was noted that 56% of the students believed they had sufficient knowledge about acne, with newspapers being the most common source of information. Hormones were identified as the most believed cause of acne, while stress was seen as the most aggravating factor. For 46% of the students, acne had little impact on their self-image, and for 73%, it had a minimal effect on their relationships [10].

A study was conducted by Alajlan et al. to estimate the prevalence of acne, assess knowledge about the condition, and explore lifestyle associations among medical students at King Saud University, Riyadh, Saudi Arabia. A total of 375 medical students participated in the study, with over half (55.5%) found to have acne vulgaris. Less than one-third of these students were diagnosed by a physician. It was observed that 61% of female students considered acne a significant medical condition, compared to 34% of male students. Additionally, 72% of female students believed that a stressful environment significantly contributed to acne vulgaris, compared to 41% of male students. Furthermore, 44.8% of male students believed that acne affected their marriage prospects, compared to 31% of female students [11].

A study was conducted by Alshehri et al. among university students in Saudi Arabia. The study aimed to evaluate the knowledge and attitudes of students towards OTC and prescription medications for acne. The study reached 420 validated responses. It was found that 52.4% of the students had used some form of OTC medication at least once, 25.7% had used prescription medications, and 21.9% had used both. Cleaners were the most commonly used OTC products, reported by 41.9% of participants. Among prescription medications, topical and oral antibiotics were the most frequently used, accounting for 11.4% of the responses. The results revealed that females were more likely to use OTC medications compared to males [12].

Allayali et al. conducted a cross-sectional study aiming to evaluate the prevalence of acne vulgaris among medical students in Saudi Arabia, as well as to assess their knowledge, attitude, and the psychological impact of acne. A total of 555 medical students’ responses were collected. The participants have consisted of 49.5% males and 50.5% females, with more than half aged between 18 and 20 years. The majority were single. Approximately two-thirds of the students were in their fourth year of medical school or earlier. Among the students, 55% had acne, with the face being the most commonly affected area and the chest the least affected area. More than half of the students used medical lotions to treat acne, while only one-third used medications. Adverse psychological effects were reported by 72.1% of the students with acne. Hormones were identified by most students as the primary aggravating factor, followed by stress, dust and heat, cosmetics, and lack of skincare and diet. Regarding knowledge about acne, the majority of male students had poor knowledge, while female students had better knowledge [13].

A study by Alraddadi et al. was conducted to determine the prevalence of acne vulgaris among medical students at Tabuk University and to explore its association with lifestyle factors such as stress, sleep deprivation, and dietary habits. A total of 301 medical students participated in the study. The prevalence of acne vulgaris was found to be 71.1%, with 42.2% of students reporting current acne and 28.9% reporting past acne. Less than half (45.3%) sought medical consultation for treatment. The severity of acne was categorized as mild (41.1%), moderate (51.9%), and severe (7%). Hormonal changes, sleep deprivation, or a combination of factors were identified as the most aggravating factors (74.8%), while food was implicated in 32.9% of cases. Associations were found between acne development and female gender, oily skin type, infrequent use of face cleanser (only once a month), and moderate to major stress [14].

Discussion

This study assesses the prevalence of self-acne treatment among medical field students at Qassim University, Saudi Arabia, and observes the self-medication of acne cases among this population group. The participation in terms of gender was equal between both males and females (50% for each group), which indicates that acne is a common concern among both genders, although a higher prevalence of acne was observed among females while assessing the association between gender and engaging in self-treatment of acne (p = 0.003) [15]. Regarding the age group, the majority of participants were young adults (19-23 years) (75%), which corresponds with the peak prevalence period of acne. Dabash et al. found that self-medication practices were common among medical students whose mean age was 21.3 years, highlighting that acne is prevalent in this age range [16].

The study revealed that a significant proportion of participants were in the clinical phase of their education (47.8%), followed by pre-clinical (25.6%) and internship (12.8%) phases. Pharmacy and dental students/interns represented a smaller percentage (8.9% and 4.4%, respectively). While the percentage of medical students who reported self-treatment of acne was 63.3%, despite their medical or healthcare-related education, the high rate of self-treatment suggests that even students with some medical knowledge may not always seek professional advice for acne [16]. On the other hand, 100% of pharmacy students in this study were engaged in practicing self-treatment of acne, which could be related to their extensive knowledge of pharmacology and easy access to medications [17].

The majority of participants (38.9%) reported acne on the face, which is consistent with studies indicating that the face is the most common site for acne due to its high density of sebaceous glands [18]. A smaller proportion reported acne on the back (1.66%) or a combination of face and back (7.8%). This is similar to findings in a study by Tan et al., where facial acne was predominant, followed by back acne [19]. The combination of face, back, and chest acne was reported by 13.3% of participants, which is consistent with research showing that acne often affects multiple sites simultaneously [20]. Notably, no participants reported acne exclusively on the chest, which contrasts with some studies where chest acne was observed, albeit less frequently than facial or back acne [21].

The most commonly used medications were topical tretinoin and benzyl peroxide, with 49 and 47 students using them, respectively. This is consistent with studies showing that topical retinoids and benzoyl peroxide are first-line treatments for acne due to their efficacy and availability [22]. Doxycycline, topical azelaic acid, and isotretinoin were each used by 24 students, which aligns with research indicating that oral antibiotics and isotretinoin are commonly prescribed for moderate-to-severe acne [23]. Topical adapalene was chosen by 22 students, reflecting its popularity as a retinoid alternative [24]. Topical clindamycin was used by 19 students, consistent with its role as a topical antibiotic in acne management [25]. Anti-androgens (spironolactone) were the least used, with only one student reporting its use, which is expected given its specific use for hormone-mediated acne in females [26].

Among the respondents, 78 participants reported using only a single medication, while 55 participants used two or more medications. This is consistent with studies showing that combination therapy is recommended for better outcomes [27]. Additionally, 47 participants did not select any medication, which may reflect reliance on non-pharmacological treatments or lack of access to medical care.

Cleansers were the most frequently used OTC treatment, with 108 students reporting their use. This is consistent with studies showing that cleansers are widely used among university students as the most common OTC acne management. Leave-on products were the next most common, used by 42 students, which aligns with the popularity of leave-on treatments such as gels and creams for their sustained efficacy. Vitamins were used by 26 students, reflecting the growing interest in dietary supplements for skin health, specifically the benefits of vitamin C in minimizing scar formation [12]. Home remedies and herbal remedies were used by 24 students, which highlights some interest in using traditional and natural remedies in acne management, which have been mentioned as potential therapy for mild-to-moderate acne cases, especially in patients who refuse drug-based treatments [28]. Essential oils were used by 18 students, who have an increasing use of aromatherapy in skincare [29]. Mechanical treatments (16 students) and peels (15 students) were less commonly used, which is consistent with their more specialized and potentially invasive nature or the need to be combined with other treatments [30].

Among the participants, 65 students reported using only a single OTC medication, while 75 students used two or more medications. This is consistent with studies showing that combination OTC treatments are often used to target multiple aspects of acne [31]. Additionally, 40 students did not select any OTC medication, which may reflect reliance on prescription treatments or non-pharmacological approaches and refers to the awareness of their cases and the proper needed treatment [32].

The most common reason for self-treatment of acne among medical students is the perception that the disease severity is mild, with 23.3% of respondents citing this reason. This suggests that many students may not consider their acne to be severe enough to warrant professional medical attention, possibly due to a lack of awareness about the potential long-term effects of untreated acne. Easy accessibility of OTC treatments was another significant factor, with 20% of respondents indicating this as their reason for self-treatment. This highlights the importance of the availability of these treatments and convenience in the decision-making process for self-medication. Around 8.3% of respondents mentioned that they knew the treatment prescribed previously, which implies that past experiences with healthcare providers influence their current self-treatment practices. Also, this could be due to a preference for familiar treatments or a belief in the effectiveness of previously prescribed medications. Embarrassment when discussing symptoms was a less common reason, with only 1.7% of respondents indicating this as a factor. This low percentage might reflect a growing acceptance and openness about discussing acne among peers and healthcare professionals. Time constraints were a factor for 5.6% of respondents, suggesting that busy schedules and the demands of medical school may lead students to opt for self-treatment as a quicker solution. Pharmacology knowledge was a significant reason for 15% of respondents, indicating that their understanding of medications and treatments plays a crucial role in their decision to self-treat. This knowledge might empower them to choose appropriate treatments confidently [8,13].

The most frequently cited sources of information were drug advertisements and social media, accounting for 23.3% of responses. This aligns with a study that identified the internet and social media as primary sources of information for self-medication among students, highlighting the significant role these platforms play in shaping health behaviors [33]. Decision-making was the next most common source, reported by 16.7% of participants. This suggests that a considerable number of students rely on their judgment when choosing acne treatments. Similarly, a study conducted in India found that 23.1% of students practiced self-medication based on their own decisions, indicating a trend of self-reliance in managing acne [34]. Family and friends, as well as pharmacists, each accounted for 12.2% of the information sources. This indicates that personal networks and healthcare professionals are common sources of advice for self-medication practices. Books were utilized by 9.4% of students as a source of information. This reflects a reliance on traditional educational resources, which, while less immediate than digital media, continue to serve as valuable references for medical students [35]. A small percentage of students (2.2%) used prescriptions written for others, and 1.7% used leftover drugs. These practices raise concerns about the appropriateness and safety of using medications not specifically prescribed for the individual, as highlighted in studies discussing the risks associated with such behaviors [9].

Regarding self-picking of the acne pimples, 94 (52.2%) of medical students reported engaging in self-picking or squeezing of acne lesions. Behaviors such as picking, scratching, squeezing, or scooping can complicate the condition and lead to acne scars, infection, and cause greater damage to the skin [9]. Despite the high prevalence of self-picking, a significant majority, 144 (80.0%) of participants did not believe that squeezing acne lesions is necessary, which indicates an awareness of the potential negative consequences associated with this practice.

In terms of exploring the statement behind engaging in self-picking of acne pimples, 37 (20.6%) of students expressed difficulty in stopping the picking or squeezing of their acne once initiated. This compulsive behavior is characteristic of excoriation (skin-picking) disorder, which has a prevalence among adolescents and young adults. The disorder often arises after the onset of dermatological conditions such as acne and can lead to significant distress and impairment. Comprehensive treatment approaches, including behavioral therapies, are recommended to address these compulsive behaviors [36]. Furthermore, 26 (14.4%) participants reported engaging in skin-picking behaviors when feeling self-conscious about their appearance. This psychological distress can manifest in compulsive skin manipulation, leading to a cycle of worsening skin condition and increased self-consciousness [37]. Additionally, 19 (10.6%) students indicated a tendency to squeeze or pick their acne pimples when under stress. Stress has been identified as a significant factor influencing acne exacerbation and related behaviors [14]. While 11 (6.1%) participants admitted to picking or squeezing their acne pimples until bleeding occurred, such actions can lead to significant skin damage and increase the risk of secondary infections. It is important to define severe cases that end up in skin and soft tissue damage as “obsessive compulsive and related disorders,” as these cases often associate with other psychiatric conditions and needs programed management including treatments of the lesions, treating the underlying psychiatric illness, and using non-pharmacological treatments such as behavioral therapy, habit reversal exercises, and support groups [38].

The majority of medical students demonstrated a good understanding of the dosage of the drug, with 50.6% indicating they knew the correct dosage. This suggests that a significant portion of students are well-informed about how much medication to take, which is crucial for effective and safe self-treatment. Regarding the mode of action of the drugs, 48.3% of respondents were aware of how the medications work. This knowledge is important as it helps students choose the right treatment based on the specific action of the acne drug. A high percentage of students, 52.2%, were knowledgeable about adverse drug reactions. This awareness is essential for recognizing potential side effects and taking appropriate measures to mitigate them. When it comes to precautions for use, 61.1% of respondents knew the necessary precautions. This indicates that many students are cautious and take steps to ensure the safe use of medications. Finally, 53.3% of students were aware of possible complications associated with self-medication. This knowledge is vital for preventing and managing any adverse outcomes that may arise from self-treatment [39].

The majority of medical students, 67.8%, did not use isotretinoin for self-treatment of acne. This could be due to the drug's potential side effects and the need for medical supervision when using it. Isotretinoin is known for its efficacy in treating severe acne, but it also carries risks such as depression, which might deter students from using it without professional guidance. Around 17.8% of respondents used isotretinoin for self-treatment. A notable 14.4% of respondents did not specify whether they used isotretinoin or not. On the other hand, all students who reported using isotretinoin had conducted the necessary laboratory tests prior to initiation, suggesting that while the medication was prescribed through regulated channels, some students perceived their use as independent or self-directed rather than under consistent medical follow-up [40].

Regarding the students’ attitude and practice towards self-medication for acne, encouragement from friends and family to self-medicate was reported by 33.3% of respondents. This indicates a significant portion of medical students believe in the efficacy and safety of self-medication, potentially influencing their social circles to adopt similar practices [41]. Consulting a dermatologist for acne was deemed necessary by 80.6% of respondents. This high percentage suggests that despite the prevalence of self-medication, students recognize the importance of professional medical advice for managing acne effectively [42]. The importance of follow-up for acne was acknowledged by 79.4% of respondents. This reflects an understanding of the chronic nature of acne and the need for ongoing monitoring and treatment adjustments. Reading drug labels and checking the expiration date regularly was a practice followed by 60.6% and 50.6% of respondents, respectively. These habits are crucial for ensuring proper use, avoiding potential misuse or adverse reactions, and ensuring the effectiveness and safety of the drugs being used. Availability of medications at home/hostel was reported by 36.7% of respondents. This indicates that a significant number of students keep medications readily accessible, which might contribute to the ease of self-treatment. While self-medication as a component of self-care was agreed upon by 68.3% of respondents, this suggests that many students view self-medication as a responsible and proactive approach to managing their health.

Limitations

This study has several limitations that should be acknowledged. First, as a cross-sectional survey conducted through social media, participants with stronger opinions or experiences regarding acne self-treatment may have been more likely to respond. Second, the use of a self-administered online questionnaire relies on the honesty and recall accuracy of respondents, particularly in reporting sensitive behaviors such as the use of prescription medications without consultation. Third, the study was conducted within a single university (Qassim University), which may limit the generalizability of the findings to broader populations. Additionally, since the sample consisted mainly of young adults aged 19-23 years, the results may not reflect the attitudes and practices of older students or non-student populations. Fourth, since the questionnaire was distributed via open online platforms, the total number of individuals who had access to or viewed the survey link could not be determined. Therefore, it was not possible to calculate a conventional response rate. Fifth, the study employed a non-random convenience sampling strategy; the findings may be subject to sampling bias, which could limit the generalizability of the results. Lastly, no qualitative data were collected to explore in depth the underlying motivations or psychosocial factors influencing self-medication behaviors, which could provide a richer context for the quantitative findings.

## Conclusions

In conclusion, this study highlights the significant prevalence of self-treatment of acne among medical students at Qassim University, Saudi Arabia. Despite their medical background, a substantial proportion of students engaged in self-medication practices, often relying on OTC products and personal knowledge rather than professional consultation. The study underscores that factors such as perceived mildness of acne, easy accessibility of medications, prior prescriptions, and pharmacological knowledge play critical roles in students’ decisions to self-medicate. Additionally, the study reveals that acne self-picking is a widespread concern, with many students exhibiting compulsive behaviors that may lead to worsening skin conditions, scars, and psychological distress.
